# Philadelphia Letter

**Published:** 1894-10

**Authors:** 


### Correspondence.

### PHILADELPHIA LETTER.

[*Special correspondence of the Buffalo Medical and Surgical Journal.*]

*Has Philadelphia Immoral Massage Establishments?—A New Medical Journal Announced on a Unique Plan—Improvements at the Jefferson Hospital—Report of the Pennsylvania Hospital and the Nativity of its Inmates—Statistics of the Insane Department—The Pennsylvania Hospital and Grounds—Nurses' Home at the Blockley Hospital—Hospital of the Medico-Chirurgical College Partially Rebuilt.*

THE feminine portion of those who advertise in the newspapers as competent to give massage treatment has recently received a blow in this city, from which recovery will not be very rapid, and a masseuse, unless she is recommended by a physician, is apt to be looked upon with suspicion. It had been noticed for some time that a series of rather striking advertisements appeared in the daily papers, for instance: A Spanish lady gives massage treatment; Massage and bath given by a young widow and assistants; Bath and massage in a first-class house by lady attendants; and so forth—a long list. Of course, everything is pure to the pure, but doctors and newspaper men have often demonstrated the fallacy of this convenient old proverb, and a reporter made the rounds of six of these massage establishments, with the result that the police made a very undignified entrance into these "parlors" the next day and arrested and fined the "attendants" as keepers of disorderly houses. And yet some call Philadelphia *slow!*

There are about twenty weekly and monthly medical journals edited and published in Philadelphia, but, to satisfy a "long-felt want," another monthly is to make its appearance soon, and it is to be run on a new plan. Every contributor must be a subscriber and every subscriber must be a contributor; and each subscriber is expected to furnish from one to three articles annually for publication and each article is to be limited to 400 or 500 words. Certainly new lines these, but the newest, probably, is that this journal-that-is-to-be needs only 200 subscribers, at $3.00 per annum, to guarantee all running expenses of publishing thirty original articles each month, illustrated, if necessary, by photo-engravings. The journal is to be very select and not in any way influenced by commercial or manufacturing concerns. A publication of that kind, with so many novel features, *ought* to succeed, and when it is in good running order I hope the Buffalo MEDICAL AND SURGICAL JOURNAL will send a representative down here to learn how an *independent* journal with *200 subscribers*, and what little advertising it can get with such a limited circulation, can live and pay its running expenses. Such remarkable financiering should not be hidden under a bushel.

The Jefferson Hospital has been thoroughly overhauled during the past Summer, disinfected, and its sanitary arrangements are now said to be perfect. It needed it badly, but its dingy, cheerless interior is now as pleasant and cherful as any modern hospital can be. Since June it has held no patients and only one room had been reserved for emergency cases ; all the others had been given over to plumbers, painters and carpenters, and next year's report of the hospital will, no doubt, show the good effect of this Summer's work in the decreased mortality of the patients.

Hospital reports are usually dry reading for the average medical man ; but once in a while we strike an item that seems to furnish food for thought to all. For instance, the report of the Pennsylvania Hospital gives a table exhibiting the number and nativity of patients received during ten years, as follows :

| | United States. | Ireland. | Russia. | Other Countries. |
|---|---|---|---|---|
| 1885 | 921 | 593 | 13 | 574 |
| 1886 | 973 | 543 | 18 | 590 |
| 1887 | 1,002 | 595 | 28 | 654 |
| 1888 | 1,085 | 555 | 41 | 684 |
| 1889 | 1,032 | 477 | 79 | 613 |
| 1890 | 1,059 | 432 | 83 | 651 |
| 1891 | 1,087 | 429 | 113 | 667 |
| 1892 | 924 | 339 | 166 | 580 |
| 1893 | 1,028 | 359 | 203 | 550 |
| 1894 | 1,012 | 325 | 251 | 621 |

Note how nearly constant is the number of native patients, as well as those from all other countries and the tremendous *increase* of sick Russians and the *remarkable* decrease of sick Irishmen. We may explain the Russian figures by the largely increased immigration from that country, but the changes in the immigration from Ireland will not explain the steady decrease in the number of sick Irishmen, and the question may be asked : Has the Irishman's health improved in a general way, or is he learning to take better care of himself when in this country? It cannot be a question of fees and money, because the hospital is practically a free institution and had during the past year an income from endowments amounting to about $375,000, while the income derived from fees of patients was only $3,000.

In a similar way the insane department of the hospital has a few very interesting items in the report. Since the formation of this department, in 1841, there have been received 10,562 patients. Of this number were by birth: Irish, 1,246; German, 578; English, 97; Scotch, 68; French, 31; Cuba, 22; and Russia only 2. With a few odd exceptions, the rest were Americans. Now, the immigration from Ireland and Germany has not differed so very much in numbers, and yet the insanity in the former is more than double that of the latter, while Russia, with its large sick list, furnishes only *two* for the insane department. It cannot be the difference between whisky and beer, because, among the whole list of 10,562 patients, only 975 are reported as insane on account of intemperance; certainly somebody with a statistical turn of mind could write a very interesting dissertation on this subject.

We of today are apt to flatter ourselves that we know all about hospital building, but the Pennsylvania Hospital, although built on its present site in 1751, shows a few points well worth applying to our up-to-date buildings. Of course, when started, it was well out of the city, and the lands surrounding the hospital were its property and used as pasture and farm lands. As the city grew, these lands were demanded for building lots, and in the early part of this century they were placed on the market. The hospital and grounds now occupy one block, but all the four surrounding blocks have been built up under very strict and stringent regulations advocated by Benjamin Franklin, who was on the board of managers. The buildings are limited to certain heighths, and, again, some of the houses have large yards or gardens on which they are *not* allowed to build at all! These "open spaces" are found on all four sides of the hospital, and ensure a better circulation of air than could have been obtained if the grounds had been surrounded by high buildings without a break excepting the street crossings. With the hot Summer here and the humid atmosphere, narrow streets and almost total absence of wind, these building regulations must inspire the visitor with admiration for the sanitary wisdom thus exhibited almost a century ago.

The Blockley Hospital is erecting a nurses' home, at a cost of $35,000. It will have accommodations for 120 nurses, and will be as perfect in its appointments as modern science can make it.

The hospital of the Medico-Chirurgical College has been partially rebuilt, and will, when completed, hold 180 beds and fifty private rooms. J. P.

